# Inflammation, Oxidative Stress, and Obesity

**DOI:** 10.3390/ijms12053117

**Published:** 2011-05-13

**Authors:** Alba Fernández-Sánchez, Eduardo Madrigal-Santillán, Mirandeli Bautista, Jaime Esquivel-Soto, Ángel Morales-González, Cesar Esquivel-Chirino, Irene Durante-Montiel, Graciela Sánchez-Rivera, Carmen Valadez-Vega, José A. Morales-González

**Affiliations:** 1 Instituto de Ciencias de la Salud, Universidad Autónoma del Estado de Hidalgo, Ex-Hacienda de la Concepción, Tilcuautla, 42080 Pachuca de Soto, Hgo, Mexico; E-Mails: alba_mfs@hotmail.com (A.F.-S.); eomsmx@yahoo.com.mx (E.M.-S.); mirandeli@hotmail.com (M.B.); m.valadezvega@lycos.com (C.V.-V.); 2 Facultad de Odontología, Universidad Nacional Autónoma de México (UNAM), México, D.F., Mexico; E-Mails: jaime_esquivel2003@hotmail.com (J.E.-S.); cesquivelch@gmail.com (C.E.-C.); 3 Escuela Superior de Cómputo, Instituto Politécnico Nacional, México, D.F., Mexico; E-Mail: anmorales@ipn.mx (A.M.-G.); 4 División de Estudios de Posgrado, Facultad de Medicina, Universidad Nacional Autónoma de México (UNAM), Mexico; E-Mail: durante@unam.mx (I.D.-M.); 5 Carrera de Médico Cirujano, FES-Iztacala, Universidad Nacional Autónoma de México (UNAM), Mexico; E-Mail: graciela_sanchez@hotmail.com (G.S.-R.)

**Keywords:** obesity, reactive oxygen species, adipokines

## Abstract

Obesity is a chronic disease of multifactorial origin and can be defined as an increase in the accumulation of body fat. Adipose tissue is not only a triglyceride storage organ, but studies have shown the role of white adipose tissue as a producer of certain bioactive substances called adipokines. Among adipokines, we find some inflammatory functions, such as Interleukin-6 (IL-6); other adipokines entail the functions of regulating food intake, therefore exerting a direct effect on weight control. This is the case of leptin, which acts on the limbic system by stimulating dopamine uptake, creating a feeling of fullness. However, these adipokines induce the production of reactive oxygen species (ROS), generating a process known as oxidative stress (OS). Because adipose tissue is the organ that secretes adipokines and these in turn generate ROS, adipose tissue is considered an independent factor for the generation of systemic OS. There are several mechanisms by which obesity produces OS. The first of these is the mitochondrial and peroxisomal oxidation of fatty acids, which can produce ROS in oxidation reactions, while another mechanism is over-consumption of oxygen, which generates free radicals in the mitochondrial respiratory chain that is found coupled with oxidative phosphorylation in mitochondria. Lipid-rich diets are also capable of generating ROS because they can alter oxygen metabolism. Upon the increase of adipose tissue, the activity of antioxidant enzymes such as superoxide dismutase (SOD), catalase (CAT), and glutathione peroxidase (GPx), was found to be significantly diminished. Finally, high ROS production and the decrease in antioxidant capacity leads to various abnormalities, among which we find endothelial dysfunction, which is characterized by a reduction in the bioavailability of vasodilators, particularly nitric oxide (NO), and an increase in endothelium-derived contractile factors, favoring atherosclerotic disease.

## 1. Introduction

Obesity is a chronic disease of multifactorial origin that develops from the interaction of social, behavioral, psychological, metabolic, cellular, and molecular factors [1]. It is the condition under which adipose tissue is increased and can be defined as an increase in body weight that results in excessive fat accumulation. The World Health Organization (WHO) defines obesity as a body mass index (BMI) > 30 and defines overweight as with a BMI of 25 [2].

## 2. Etiological Factors

Fundamentally, obesity is the result of excessive energy consumption compared with the energy expended; in children, increased consumption of fats and sugars and lack of physical activity have been linked with obesity [2].

The basic hypothesis of the cause of the disease is the existence of the “thrifty gene theory”, which suggests that some populations may have genes that determine increased fat storage, the latter when experiencing periods of starvation, thus providing a survival advantage; but under current circumstances, overstocking of fat results in obesity and Type 2 diabetes mellitus (T2DM) [2]. On the other hand, it has been postulated that in the early stages of our evolution, highly effective systems were developed to collect the limited energy available, leading to the appearance of adipose tissue. The lack of industrial development meant long hours of exhaustive physical exercise to achieve limited amounts of food. This energy would accumulate efficiently for later use.

Changes in lifestyle and diet have resulted in an increase in the number of obese subjects; obesity has been regarded as an important factor in causing various health problems [3]. Another theory for explaining the development of obesity is known as the fetal origins hypothesis of chronic diseases. This suggests that poor maternal nutrition and poor fetal growth are risk factors for developing chronic diseases that affect the programming of body structure, physiology, and metabolism [4]. The central nervous system (CNS), by means of signals, regulates appetite, energy intake, and weight gain; obesity can result from a failure of these signaling pathways [2].

## 3. Epidemiology

Obesity is considered the largest public health problem worldwide, especially in industrialized countries [5]. Obesity increases mortality and the prevalence of cardiovascular diseases, diabetes, and colon cancer [6]. Substantial literature has emerged that shows that overweight and obesity are major causes of co-morbidities, including T2DM, cardiovascular diseases, various cancers, and other health problems, which can lead to further morbidity and mortality. The related health-care costs are also substantial. Therefore, a public health approach to develop population-based strategies for the prevention of excess weight gain is of great importance. However, public health intervention programs have had limited success in tackling the rising prevalence of obesity. This paper reviews the definition of overweight and obesity and variations regarding age and ethnicity and health consequences and factors contributing to the development of obesity, and presents a critical review of the effectiveness of current public health strategies for risk factor reduction and obesity prevention [7].

## 4. Adipose Tissue

Human adipose tissue is divided into brown adipose tissue, which possesses multilocular adipocytes with abundant mitochondria that express high amounts of uncoupling protein 1 (UCP-1), which is responsible for the thermogenic activity of this tissue [8], and white adipose tissue, which is responsible for fat storage. Among the characteristics of white adipose tissue, we found that it consists of different cell types such as fibroblasts, preadipocytes, mature adipocytes, and macrophages. This tissue is very heterogeneous according to its visceral or subcutaneous location [9].

**Figure 1.**
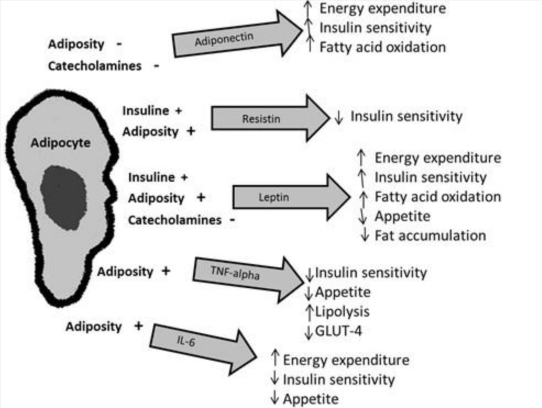
This figure depicts the major adipokines and their roles. Adipose tissue produces several adipokines that exert metabolic effects, both in central and in peripheral tissues. The production of these adipokines is regulated by insulin, cathecholamines, and adiposity. TNF-alpha: Tumor necrosis factor-alpha; IL-6: Interleukin. (*Courtesy of Cristina Fernández-Mejía, Ph.D.*).

In animals with obesity, there is a huge increase in white fat deposits due to the hyperplasia and hypertrophy of their adipocytes [8]. Hypertrophic-hyperplastic adipocytes exhibit a lower density of insulin receptors and a higher beta-3 adrenergic receptor, which facilitates the diapedesis of monocytes to visceral adipose stroma, initiating a proinflammatory cycle between adipo- and monocytes [10]. Adipose tissue is not only a triglyceride (TG)-storage tissue; studies in recent years have shown the role of white adipose tissue as a producer of certain substances with endocrine, paracrine, and autocrine action [2]. These bioactive substances are denominated adipokines or adipocytokines, among which are found plasminogen activator inhibitor-1 (PAI-1), tumor necrosis factor-alpha (TNF-α), resistin, leptin, and adiponectin [11]. These substances derive primarily from white adipose tissue and play a role in the homeostasis of various physiological processes (Figure 1).

## 5. Adipokines and Metabolic Homeostasis

### 5.1. Leptin

Leptin was discovered in 1994. It is a hormone secreted mainly by adipocytes in direct proportion to the mass of adipose-tissue TG content and the nutritional condition [3]. In order to be secreted by adipose tissue, leptin circulates in plasma bound to plasma proteins, entering by diffusion into the CNS, through capillary binding in the median eminence and by saturable transport across the choroid plexus receiver. In the ventro-medial nucleus of the hypothalamus, leptin stimulates cytokine receptor kinase 2 (CK2), the synthesis of melanocyte-stimulating hormone and by cocaine-amphetamine-regulated transcriptor (CART) molecules that, via paracrine, stimulate receptors 3 and 4 of the lateral melanocortin nucleus, causing satiety [2,11].

Leptin inhibits lipogenesis and stimulates lipolysis, reducing intracellular lipid levels in skeletal muscle, liver, and pancreatic beta cells, thereby improving insulin sensitivity. The limbic system stimulates dopamine reuptake, thereby blocking the pleasure of eating and, through the locus coeruleus nucleus, activates the sympathetic nervous system, which leads to increased resting energy expenditure [11].

Catecholamines influence the production of leptin, and other leptin production regulators comprise the glucocorticoids [2], although it has been postulated that the main determinant of leptin secretion is glucose metabolism, because the concentration of circulating leptin diminishes under fasting or caloric restriction conditions and increases in response to food intake [8]. Obesity is associated with increased leptin levels; as a result, it has been postulated that the apparent decrease in anorexigenic effects and weight loss are the result of a mechanism of resistance to it [11].

In inflammation, leptin acts directly on macrophages to increase phagocytic activity, and proinflammatory cytokine production also exerts an effect on T-cells, monocytes, neutrophils, and endothelial cells. When leptin is administered, increased levels of C-reactive protein (CRP) are produced, thus proving its inflammatory effect [12]. When there is weight loss, circulating levels of the hormone are reduced, and in turn, these levels reduce the plasma levels of obesity-associated inflammatory markers [13].

In addition to promoting oxidative stress and vascular inflammation, leptin stimulates proliferation and migration of endothelial cells and smooth muscle cells, thus favoring the development of atherosclerosis [14]. It is noteworthy that leptin can also be produced in placenta, spinal cord, stomach, muscle, and perhaps in brain, which increases the regulatory role of this hormone [2].

### 5.2. Tumor Necrosis Factor Alpha (TNF-α)

TNF-α was one of the first cytokines identified and is involved in the systemic inflammatory response; additionally, it has also has been linked with the development of insulin resistance, obesity, and diabetes [9]. It is produced mainly by monocytes, lymphocytes, adipose tissue, and muscle [15] and its irregular production participates in the pathogenesis of the obesity-associated metabolic syndrome.

TNF-α activity on insulin resistance can be explained as follows: it increases the release of free fatty acids (FFA) in adipocytes; it blocks the synthesis of adiponectin, which possesses insulin-sensitizing activity in high concentrations in adipose tissue, and it interferes with the activity of tyrosine-residue phosphorylation activity in the first substrate of the insulin receptor, which is necessary for progression of the intracellular signal of the hormone [3]. The TNF-α activates nuclear factor κB (NF-κ B), resulting in the increased expression of adhesion molecules on the surface of endothelial cells and vascular smooth muscle cells, resulting in an inflammatory state in adipose tissue, endothelial dysfunction, and, ultimately, atherogenesis [3].

### 5.3. Interleukin 6 (IL-6)

This is a cytokine that exerts many effects, ranging from defense to inflammation and tissue damage [11]. It is produced both by macrophages and adipocytes [14], and by immune system cells, fibroblasts, endothelial cells, and skeletal muscle [9]. Circulating levels of IL-6 correlate with BMI, insulin resistance, and intolerance to carbohydrates [3]. IL-6 also influences glucose tolerance through negative regulation of visfatin; in addition, it antagonizes the secretion of adiponectin [11], and in animal model, it elevates TG levels by enhancing gluconeogensis and glycogenolysis and inhibiting glycogenesis.

### 5.4. Angiotensinogen/PAI-1

Angiotensinogen may play an important role in the regulation of adipose tissue blood supply and the flow of fatty acids from the same [8]. It is expressed in multiple cell types within adipose tissue; its expression and secretion are higher in visceral tissue than in subcutaneous tissue, and its high levels correlate with metabolic syndrome [11].

Plasminogen activator inhibitor (PAI-1) is the first physiological inhibitor of plasminogen activators in the blood and contributes to thrombus formation and the development of chronic cardiovascular disease. PAI-1 can play an important role in the regulation of adipose tissue blood supply and the flow of fatty acids from it. Plasma levels of PAI-1 are regulated by the accumulation of visceral fat, and a high concentration of PAI-1 is associated with insulin resistance as well as with pro-inflammatory cytokines [2].

### 5.5. Adiponectin

Adiponectin is a protein that is structurally homologous to collagens VIII and X and of complementary system factor C1q, also known as ADIPOQ, Acrp, APM1, and GBP. Adiponectin expression and secretion is unique to differentiated adipocytes [8] and has regulatory actions on energy homeostasis, glucose and lipid metabolism, and anti-inflammatory action [2]. In contrast to other adipokines, adiponectin expression and plasma concentrations are not increased, but are rather decreased in a wide variety of diseases presenting insulin resistance and obesity [9]. High levels of this adipokine are related with weight loss [2] and, in addition, adiponectin improves insulin sensitivity, decreases the flow of free fatty acids and increases their oxidation, inhibits major gluconeogenic liver enzymes, reduces hepatic release of glucose and muscle, and stimulates glucose utilization and fatty acid oxidation [3]. Adiponectin shows high anti-inflammatory and antiatherogenic powers because it inhibits the adhesion of monocytes to endothelial cells, the transformation of macrophages into foam cells and endothelial cell activation, inhibits TNF-α expression [2], decreases CRP levels, and increases nitric oxide (NO) production in endothelial cells [16]. Its globular isoform inhibits cell proliferation and production of ROS induced by low-density lipoprotein (LDL) oxidase during atheromatous plaque formation [17]. In general, adiponectin deficiency results in NO reduction in the vascular walls and promotes leukocyte adhesion, causing chronic vascular inflammation [16]. Finally, it was observed that TNF-α and IL-6 are potent inhibitors of adiponectin expression and secretion [9].

### 5.6. Adipsin

Adipsin is a relatively small serine protease that is secreted by adipocytes and that is positively related with adiposity, insulin resistance, dyslipidemia, and cardiovascular disease. Adipsin appears to regulate the rate at which fatty acids from Lipoprotein lipase (LPL) are taken up by adipocytes and subsequently converted into TG. The molecular basis of the pathogenesis of obesity-linked disorders has not been fully elucidated. Adipose tissue serves not only as an energy storage organ, but also as an endocrine organ. It releases many factors with autocrine, paracrine, and endocrine functions. Adipokines such as adipsin are biologically active molecules produced by adipose tissue. They play a role in energy homeostasis, and in glucose and lipid metabolism [18].

### 5.7. Resistin

Resistin (RSTN) is an adipokine produced by mature adipocytes and macrophages, and it has been postulated that resistin might comprise the link between obesity and insulin resistance [8]. This adipokine belongs to the family of secreted proteins termed cysteine-rich Found in inflammatory zone (FIZZ), and the approved gene symbol is *RETN*. It possesses hyperglycemic properties; circulating resistin levels are proportional to the degree of adiposity but are not related with the degree of insulin resistance [9]. RSTN is a link to the inflammatory environment due to its predominant production of monocytes and its correlation with IL-6 levels [11].

On the other hand, Type 2 diabetes mellitus (T2DM), characterized by target-tissue resistance to insulin, is epidemic in industrialized societies and is strongly associated with obesity; however, the mechanism by which increased adiposity causes insulin resistance is unclear. Adipocytes secrete a unique signaling molecule, which we have denominated resistin (for resistance to insulin). Circulating resistin levels are decreased by the anti-diabetic drug rosiglitazone and are increased in diet-induced and genetic forms of obesity. Administration of the anti-resistin antibody improves blood sugar and insulin action in mice with diet-induced obesity. Moreover, treatment of normal mice with recombinant resistin impairs glucose tolerance and insulin action. Insulin-stimulated glucose uptake by adipocytes is enhanced by neutralization of resistin and is reduced by resistin treatment. Thus, resistin is a hormone that potentially links obesity with diabetes [19,20].

### 5.8. Other Adipokines

Visfatin is an important adipocytokine. Concentrations of this adipokine are increased in humans with abdominal obesity and Diabetes mellitus (DM). Its increased concentration in obesity could be a compensatory response in an attempt to maintain blood euglycemia. The regulation of its synthesis is stimulated by glucocorticoids and inhibited by TNF-α, IL-6, growth hormone, and β-adrenergic receptor agonists [21].

Visfatin stimulates adipocytes differentiation, promotes the accumulation of TG from glucose, and induces expression of genes encoding for diacylglycerol acyltransferase and for adiponectin by means of a reduction in glucose release from adipocytes [11].

Another adipokine is omentin, a peptide secreted by visceral fat and, contrary to visfatin, it appears to be produced to a greater degree in vascular stromal cells within the fat than in the adipocytes themselves. Similar to visfatin, it exerts beneficial effects on glucose uptake, functions as an insulin sensitizer, and possesses insulin-mimicking properties [22].

Finally, there is apelin, whose receptor is expressed in brain and in nearly all peripheral tissues, especially in endothelial cells in cardiac, kidney, lung, adrenal, and endocardial vessels. Apelin causes NO-mediated, endothelium-dependent vasodilation and endothelium-independent vasoconstriction by means of its action on smooth muscle cells. Apelin is produced in proportion to the amount of fat and possesses anorectic properties accompanied by increased body temperature and locomotor activity, as well as inhibiting the secretion of glucose-dependent insulin [11].

## 6. Lipotoxicity

Adipocytes of patients with obesity have a lower insulin receptor density and a higher density of beta-3 adrenergic receptors, thus increasing the lipolysis rate with release of FFA, a situation that has several metabolic consequences in which the following are present: increase in the production of oxygen-derived free radicals; induction of insulin resistance; synergism in the action of IL-6 and TNF-α, and induction of apoptosis in pancreatic beta cells; taken together, these effects are categorized as lipotoxicity. Lipotoxicity causes both anatomical and functional injury in different cell lines. Adipose tissue dysfunction as well as lipotoxicity comprise two mechanisms that explain the proinflammatory state and insulin resistance (IR) [11,23].

## 7. Obesity and Oxidative Stress

ROS occur under physiological conditions and in many diseases and cause direct or indirect damage in different organs; thus, it is known that oxidative stress (OS) is involved in pathological processes such as obesity, diabetes, cardiovascular disease, and atherogenic processes. It has been reported that obesity may induce systemic OS and, in turn, OS is associated with an irregular production of adipokines, which contributes to the development of the metabolic syndrome [24]. The sensitivity of CRP and other biomarkers of oxidative damage are higher in individuals with obesity and correlate directly with BMI and the percentage of body fat, LDL oxidation, and TG levels [25]; in contrast, antioxidant defense markers are lower according to the amount of body fat and central obesity [26,27]. A research showed that a diet high in fat and carbohydrates induces a significant increase in OS stress and inflammation in persons with obesity [28].

Pathophysiology of OS:

- Peroxisomal fatty acid metabolism, in which H_2_O_2_ is formed as a byproduct, and despite that peroxisomes contain high catalase activity, they may cause OS under certain pathological conditions.
- Cytochrome P450 microsomal reactions, which catalyze the metabolism of xenobiotic compounds by oxidoreducers, forming superoxide anion as a byproduct, which can cause OS.
- Phagocyte cells, which attack invasive pathogens with a mixture of ROS and other oxidants. This is an immune response, but also damages surrounding tissues, producing inflammation.
- The mitochondrial respiratory chain. It is considered that the mitochondria are the site within the cell where the largest amount of ROS are generated, causing defects in mitochondrial metabolism and diseases.

OS biomarkers, such as malondialdehyde (MDA) and F-2 isoprostanes (F2-IsoPs), are the products of the peroxidation of polyunsaturated fatty acids. One study showed that BMI was significantly related with the concentration of F2-IsoPs. In addition, dietary factors were analyzed, and it was observed that fruit consumption is inversely associated with the level of lipid peroxidation. This same study revealed that females demonstrated a higher peroxidation level compared with males, which may be caused by the higher percentage of fat possessed by females. We also found a positive relationship between lipid peroxidation level and plasma cholesterol concentration [29].

Another OS marker is the urinary levels of 8-iso Prostaglandin F2α (8-iso PGFα), which are positively related with obesity and insulin resistance [30] and negatively associated with plasma concentration of adiponectin.

## 8. Mechanisms of Formation of Free Radicals during Obesity

### 8.1. Adipose Tissue

The increase in obesity-associated OS is probably due to the presence of excessive adipose tissue itself, because adipocytes and preadipocytes have been identified as a source of proinflammatory cytokines, including TNF-α, IL-1, and IL-6; thus, obesity is considered a state of chronic inflammation. These cytokines are potent stimulators for the production of reactive oxygen and nitrogen by macrophages and monocytes; therefore, a rise in the concentration of cytokines could be responsible for increased OS. TNF-α also inhibits the activity of PCR, increasing the interaction of electrons with oxygen to generate superoxide anion [11]. Adipose tissue also has the secretory capacity of angiotensin II, which stimulates Nicotinamide adenine dinucleotide phosphate (NADPH) oxidase activity. NADPH oxidase comprises the major route for ROS production in adipocytes [31].

### 8.2. Fatty Acid Oxidation

Mitochondrial and peroxisomal oxidation of fatty acids are capable of producing free radicals in liver and, therefore, OS, which could result in mitochondrial DNA alterations in the oxidative phosphorylation that occurs in mitochondria, causing structural abnormalities and depletion of adenosine triphosphate (ATP). However, it is also possible that mitochondrial abnormalities are preexisting conditions that allow for overproduction of ROS [32].

### 8.3. Overconsumption of Oxygen

Obesity increases the mechanical load and myocardial metabolism; therefore, oxygen consumption is increased. One negative consequence of increased oxygen consumption is the production of ROS as superoxide, hydroxyl radical, and hydrogen peroxide derived from the increase in mitochondrial respiration and, of course, from the loss of electrons produced in the electron transport chain, resulting in the formation of superoxide radical [6,33].

### 8.4. Accumulation of Cellular Damage

Excessive fat accumulation can cause cellular damage due to pressure effect from fat cells (*i.e.,* non alcoholic steatohepatitis). Cellular damage in turn leads to high production of cytokines such as TNF-α, which generates ROS in the tissues, increasing the lipid peroxidation rate [33].

### 8.5. Type of Diet

Another possible mechanism of ROS formation during obesity is through diet. Consumption of diets high in fat may alter oxygen metabolism. Fatty deposits are vulnerable to suffering oxidation reactions. If the production of these ROS exceeds the antioxidant capacity of the cell, OS resulting in lipid peroxidation could contribute to the development of atherosclerosis [33].

### 8.6. Role of Mitochondria in the Development of OS in Obesity

Mitochondria provide the energy required for nearly all cellular processes that ultimately permit the carrying out of physiological functions; additionally, they play a central role in cell death by the mechanism of apoptosis. Mitochondrial dysfunction has been implicated in a variety of diseases ranging from neurodegenerative diseases to diabetes and aging. Obesity takes place in disorders that affect mitochondrial metabolism, which favors ROS generation and the development of OS. On the other hand, another mechanism has been proposed that involves an effect of high triglyceride (TG) on the functioning of the mitochondrial respiratory chain, in which intracellular TG, which is also high, inhibits translocation of adenine nucleotides and promotes the generation of superoxide [34].

The mitochondrial process of oxidative phosphorylation is very efficient, but a small percentage of electrons may prematurely reduce oxygen, forming potentially toxic free radicals, impairing mitochondrial function. Beyond that, under certain conditions, protons can be reintroduced into the mitochondrial matrix through different uncoupling proteins, affecting the control of free radical production in mitochondria [35]. Uncoupling proteins possess an amino acid sequence that is utilized to identify potential mitochondrial carriers. To date, three molecules have been described in mammalian mitochondria: UCP-1, -2, and -3. UCP-1 is involved in the control of adaptive thermogenesis and weight control. UCP-3, which in humans is found only in skeletal muscle, appears to exert an effect on heat issue, but protects the mitochondria of lipotoxicity in cases of increased concentrations of FFA in the matrix, because it leads these to the intermembrane space. During obesity, an increase in FFA, which is toxic to pancreatic cells that are sensitive to oxidation and inducing alterations in insulin release, may lead to the development of DM [34]. The potential roles of UCP-2 include control of ATP synthesis, regulation of fatty acid metabolism, and, thereby, control of ROS 3- production; it is also postulated that UCP-2 can mobilize the FAA outside of the mitochondrial matrix; FAA are detrimental to the proper functioning of this organelle [36].

## 9. Complications-generated Oxidative Stress in Obesity

Obesity and the consequent production of OS have been associated with the development of other pathologies (Table 1), the most straightforward of which is the metabolic syndrome.

Another of the changes related with obesity is the development of non-alcoholic steatohepatitis, which appears as a result of the increased circulating FFAs that are released by adipose tissue in response to insulin resistance. The amount of internalized FFA in liver is not regulated; thus, it is proportional to the plasma, in addition it also increases lipogenesis in the body and enhances intracellular accumulation of TG [34]. Excessive accumulation of fat (TG) in the liver is the first step in the development of non-alcoholic fatty liver disease, while the second step is inflammation and cirrhosis.

## 10. Obesity and Antioxidant Capacity

When obesity persists for a long time, antioxidant sources can be depleted, decreasing the activity of enzymes such as superoxide dismutase (SOD) and catalase (CAT) [6]. The activity of SOD and glutathione peroxidase (GPx) in individuals with obesity is significantly lower compared with that in healthy persons, having implications for the development of obesity-related health problems [37]. A study in rats showed that the liver concentration of vitamin A having antioxidant activity was significantly lower in rats with obesity compared with those without obesity; the concentration of vitamin A in rats with obesity probably indicates the dilution of this fat-soluble vitamin in high liver lipid storage [38]. In addition to vitamin A, levels of serum antioxidants, such as vitamin E, vitamin C, and β-carotene, as well as glutathione, are decreased in obesity [39]. In addition to this, ROS decrease the expression of adiponectin, suggesting that treatment with antioxidants or ROS inhibitors could restore the regulation of adipokines [40]. Thus, supplementation with antioxidants would reduce the risk of complications related with obesity and OS [41].

## 11. Nitric Oxide in Obesity

Nitric oxide (NO) is a physiological regulator of diverse functions in several tissues including cardiovascular, neuromuscular, neurological, genitourinary, gastrointestinal, and renal. Inhibitors of nitric oxide synthase (INO) reduce NO production and prevent the decrease in insulin secretion caused by free fatty acids [42]. NO is an important anti-atherogenic agent and it inhibits platelet activation and aggregation, leukocyte chemotaxis, and endothelial adhesion [43]. Endothelium-dependent vasodilation of NO is impaired under conditions of overweight and obesity, which is observed equally in the presence of hypercholesterolemia [44].

An increase in the production of superoxide as well as the expression of endothelial NO production may increase peroxynitrite in persons with obesity and high blood pressure, diminishing the availability of NO and causing vasoconstriction in the vasculature of the liver [45].

## 12. Inflammation and Obesity

This is called low-intensity chronic inflammation to the inflammatory response and it lasts several days, weeks, or months in response to the presence of foreign agents in the bloodstream; it is also defined as a protective reaction of vascular connective tissue to injurious stimuli including infection. There is a clear-sightedness not only that a low-intensity chronic inflammation co-exists, but also that it precedes the development of T2DM; one example is the presence of markers that have predictive capacity in relation to T2DM, such as PCR and IL-6. Inflammation is characterized by vasodilation, vascular permeability, and inflammatory cells such as neutrophils and cytokines [46].

Inflammation is a manifestation of increased OS, which increases in subjects with obesity and which is related with insulin resistance and endothelial dysfunction. These changes may interact among themselves and amplify, producing, in this manner, the set of metabolic and vascular alterations [11]. One possible explanation for adipose tissue producing adipokines and acute phase proteins is the consideration of hypoxia as the trigger. Hypoxia would be produced during the overgrowth of adipose tissue during obesity. Adipose tissue produces 25% of systemic IL-6; thus, it is said that this adipose tissue may induce a lesser degree of systemic inflammation in persons with excess body fat. The overall evidence indicates that, compared with macrophages, fat cells have a capacity equal to or greater than inflammatory cells, and it has been observed that the increase of the factors released by adipocytes may be reflected in systemic inflammation [47]. Nishimura *et al*. [48] suggest that obese adipose tissue activates CD8(+) T-cells, which, in turn, promote the recruitment and activation of macrophages in this tissue. These results support the notion that CD8 (+) T-cells play an essential role in the initiation and propagation of adipose inflammation. Cani *et al*. [49] demonstrated that in high-fat diet-fed mice, the modulation of gut microbiota is associated with an increased intestinal permeability that precedes the development of metabolic endotoxemia, inflammation, and associated disorders, and found that in ob/ob mice, gut microbiota determines plasma LPS concentration and is a mechanism involved in metabolic disorders.

## 13. Endothelial Dysfunction

The vascular endothelium is a paracrine, endocrine, and autocrine organ that is indispensable for the regulation of vascular tone and the maintenance of vascular homeostasis. Endothelial dysfunction is characterized by a reduction in the bioavailability of vasodilators, particularly NO, and an increase in endothelium-derived contractile factors. It also includes a specific state of endothelial activation that is characterized by a proinflammatory, proliferative, and procoagulant state, all favoring atherogenesis [50]. Endothelial dysfunction can be caused by stimulating inflammation and free radicals and cytokines; LDL oxidation is also associated with an increase in the expression of adhesion molecules in the endothelium, which facilitates monocyte infiltration into the subendothelial space [51]. The polymerase chain reaction (PCR) favors lower NO activity by increasing the production of factors that inhibit the latter’s functions, such as endothelin and angiotensin II, thereby reducing the beneficial actions exerted by NO on vascular function [14]. Recently, the role of adipose tissue and its secretory adipokine as a major cause of endothelial dysfunction has been emphasized. Adipose tissue dysfunction, as occurs in obesity and insulin resistance, is characterized by the activation of an inflammatory signal. Some of these signals arise, directly or indirectly, from substances secreted in adipose tissue. ROS are generated at sites of inflammation and damage; a high concentration of these can cause cell damage and death, and specifically, OS increases vascular endothelial permeability and promotes leukocyte adhesion [50]. During obesity, there is a higher content of superoxide radicals and nitrotyrosine in the coronary endothelium, and early obesity is characterized by increased OS and endothelial dysfunction associated with increased leptin levels [52]: in addition, it has been reported that weight loss improves endothelium-dependent vasodilatation, improves endothelial activation markers, and decreases proinflammatory cytokine levels [50].

## 14. Conclusions

Adipose tissue is a secretory organ of great importance for the organism because the substances that it secretes meet the requirements for specific biological functions. As obesity is characterized by excessive storage of adipose tissue, adipokine secretion is increased; therefore, the effects produced in the body are altered, and resistance to its effect can be generated, as in the case of leptin. In addition to adipokines, we also found an overproduction of ROS, which damage cellular structures and trigger, together with underproduction of NO, progressive accumulation of fat and, eventually, the development of other pathologies. On the other hand, it was observed that the decrease in body fat reflected in weight improves oxidation markers and increases antioxidant activity, which was impaired with obesity. Therefore, weight loss through nutritional and pharmacological treatment, in addition to supplementation with antioxidant nutrients such as vitamins E, A, and C, flavonoids, among others, may be the key to reducing the risk of developing other pathologies related with OS and obesity such as high blood pressure and, of course, metabolic syndrome.

Obesity is a condition that is epidemic and that has increased in recent decades. Parallel to the increase of this disease, the study of obesity has undergone considerable development. This has been accomplished thanks to research in various fields of knowledge that have broken down multiple archetypes, allowing changes in views on overweight, adipose tissue function, and the pathophysiology of the disease that prevail at present. The breakdown of old paradigms and the new knowledge platform provide a solid foundation for understanding the disease and for developing strategies for prevention and treatment.

## Figures and Tables

**Table 1.** Diseases associated with obesity.

Insulin resistance and diabetes
Systemic arterial hypertension
Ischemic heart diseases
Obstructive sleep apnea, asthma
Gout
Peripheral vascular disease
Psychology problems (social stigmatization)
Rheumatological and orthopedics problems
Oncology problems
Liver failure
